# Application of injectable hydrogels in cancer immunotherapy

**DOI:** 10.3389/fbioe.2023.1121887

**Published:** 2023-02-03

**Authors:** Chutong Liu, Yingying Liao, Lei Liu, Luoyijun Xie, Junbo Liu, Yumao Zhang, Yuzhen Li

**Affiliations:** The Eighth Affiliated Hospital, Sun Yat-sen University, Shenzhen, Guangdong, China

**Keywords:** injectable hydrogel, cancer immunotherapy, delivery system, chemotherapy, radiotherapy, phototherapy, DNA hydrogel

## Abstract

Immunotherapy is a revolutionary and promising approach to cancer treatment. However, traditional cancer immunotherapy often has the disadvantages of limited immune response rate, poor targeting, and low treatment index due to systemic administration. Hydrogels are drug carriers with many advantages. They can be loaded and transported with immunotherapeutic agents, chemical anticancer drugs, radiopharmaceuticals, photothermal agents, photosensitizers, and other therapeutic agents to achieve controlled release of drugs, extend the retention time of drugs, and thus successfully trigger anti-tumor effects and maintain long-term therapeutic effects after administration. This paper reviews recent advances in injectable hydrogel-based cancer immunotherapy, including immunotherapy alone, immunotherapy with combination chemotherapy, radiotherapy, phototherapy, and DNA hydrogel-based immunotherapy. Finally, we review the potential and limitations of injectable hydrogels in cancer immunotherapy.

## Introduction

As early as the 19th century, someone put forward the idea of using our immune system to classify and damage cancer cells, known as tumor immunotherapy (Wiemann and Starnes, 1994). Immunotherapy is one type of treatment that uses regulators or drugs to initiate and regulate the immune response to destroy malignant cells. Cancer immunotherapy is a revolutionary effective cancer therapy method next to traditional operation, chemotherapeutic and radiotherapeutic (Topalian et al., 2015; Nam et al., 2019). It has changed people’s way of thinking from directly destroying cancer cells to recognizing and attacking cancer cells by triggering the host’s own anti-cancer immunity. There are more than 30 different types of immunotherapies approved by FAD, mainly immunosuppressants, tumor vaccines, cytokines, chimeric antigen receptor T cell (CAR-T) therapies, and immune checkpoint blockade (ICB) (Postow et al., 2015; Riley et al., 2019a; Sterner and Sterner, 2021). Different from conventional treatment approaches, such as surgery, radiotherapy, chemotherapeutic, etc., which are merely effective for resident solid cancers, immunotherapeutic can destroy resident and remote metastatic cancers, and inhibit cancer recurrence. However, traditional cancer immunotherapy methods often have disadvantages such as expensive cost (Zhao et al., 2020), low therapeutic index, poor targeting, high drug resistance, and serious immune-related adverse events (IRAEs), which are primarily ascribed to high doses or multiple injections of immunotherapy drugs (Blattman and Greenberg, 2004; Milling et al., 2017; Senapati et al., 2018; Riley et al., 2019b; Zhao et al., 2019; Haanen et al., 2020). The traditional immunotherapy strategy is often a systemic administration of immune drugs. Unfortunately, only a small number of drugs administered intravenously can reach the target of cancer cells in their body, while the remaining circulating drugs may damage normal cells. Therefore, to improve the efficacy of each dose and reduce side effects, appropriate nanomaterials are selected as drug carriers to overcome the biological barrier and achieve enhanced uptake of immunotherapeutic agents in cancer cells (Riley et al., 2019b; Zhao et al., 2019; Yan et al., 2020). Among them, hydrogel, due to its high biocompatibility, allows for a multi-purpose design to provide both sensing and therapy (Oliva et al., 2017).

Over recent years, hydrogels have been widely studied by scholars for its excellent physical, chemical and biological performances and potential application prospect. They are highly crosslinked, water-swelling networks of hydrophilic three-dimensional polymers that retain a lot of water in its 3D network (Buwalda et al., 2014). Hydrogels formed *in situ* has recently become one of the most favorable biomedical ingredients and can be sprayed directly or delivered into the body. *In situ* hydrogels have shown several advantages, including fewer intrusive handling, easy use, effortless cell embedding, and proficient encapsulation (Liao et al., 2022). The application of hydrogels in many aspects has shown great promise. So far, it has been applied as typical biocompatible materials (Huang et al., 2017; Tong and Yang, 2018), wound repair (Liu et al., 2007; Tavakoli and Klar, 2020), drug delivery (Dimatteo et al., 2018; Mathew et al., 2018), cell therapeutic (Sivaraj et al., 2021), tumor therapeutic (Yu et al., 2018a; Bu et al., 2019) and extra areas.

Hydrogel is an appealing “intelligent” drug delivery system, which can deliver a variety of bioactive agents to specific locations, and has the ability of controllable and supportable release (Singh and Peppas, 2014; Li and Mooney, 2016). By implanting hydrogels, immunotherapy drugs are delivered in the body so that they can circulate in the blood for longer periods, accumulate effectively in tumors, increase drug concentration, and actively target cancer cells to improve the efficacy of anti-tumor immunotherapy (Jin et al., 2018). Therefore, injectable hydrogels can effectively overcome the above obstacles of systemic administration. At present, injectable hydrogels have become a significant part of tumor immunotherapy (Yu et al., 2018a).

## Injectable hydrogels for cancer immunotherapy

**FIGURE 1:**
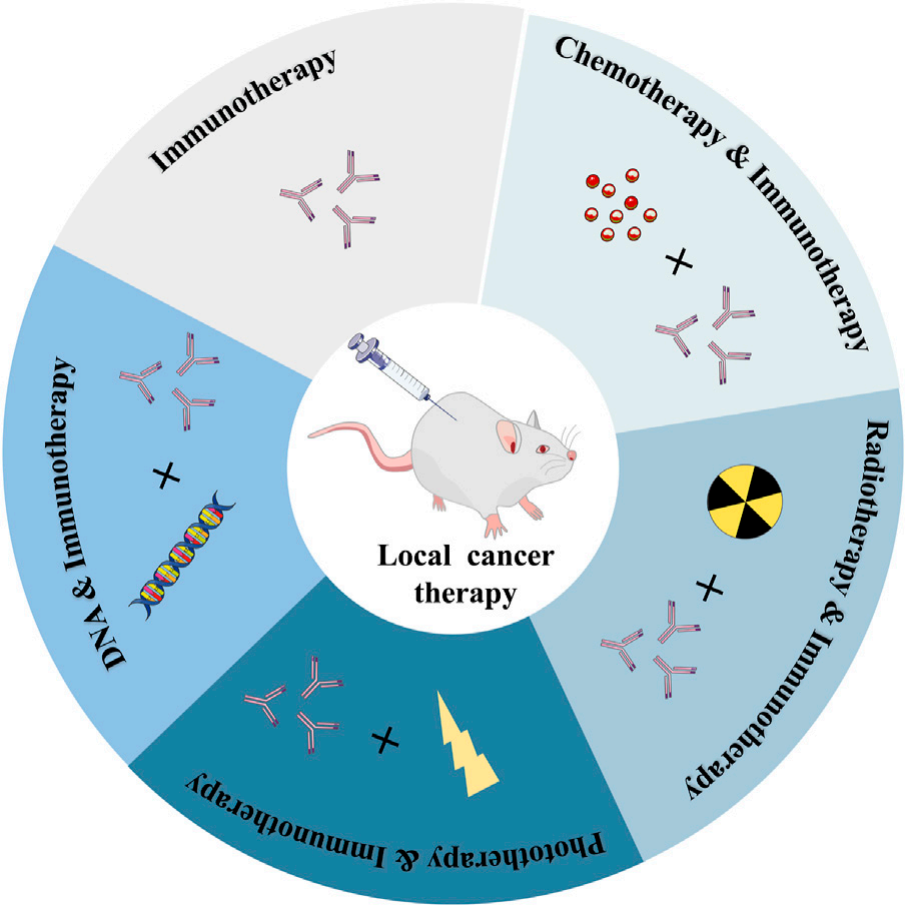
Common combined immunotherapy strategies for local cancer treatment.

Various immunotherapy drugs that can cause anti-tumor immune responses have been developed recently, but the inefficiency of drug delivery and serious side effects of these anti-cancer drugs throughout the body have hindered their use in cancer immunotherapy (Kim et al., 2022). It can specifically deliver and continuously release drugs with therapeutic effects at the target site, which not only reduces the systemic toxicity caused by systemic circulation but also improves the administration efficiency, therefore, the efficacy of cancer treatment is significantly improved (Elstad and Fowers, 2009; Wu et al., 2016; Shin and Kwon, 2017; Tiwari et al., 2018; Attama et al., 2022). What’s more, it enables the regulated release of the medicine, minimizing the non-specific distribution of the drug in healthy tissue (Hamidi et al., 2008; Li and Mooney, 2016; Xue et al., 2017; Riley et al., 2019b; Xie et al., 2021; Li Y. et al., 2022). This controlled drug delivery action based on polymer hydrogels with high spatiotemporal precision has been used in cancer immunotherapeutics (Thambi et al., 2016; Duong et al., 2020). The recent research progress of cancer immunotherapy based on injectable hydrogels, including immunotherapy alone, immunotherapy combined with chemotherapy, radiotherapy, phototherapy, and immunotherapy based on DNA hydrogel are currently being explored (Figure 1).

## Sole immunotherapy based on hydrogel delivery

Immunotherapy drugs have presented huge promise for cancer, however, their widespread application in clinical practice is challenging (Zhao et al., 2019; Xie et al., 2021). Lately, the local administration of immunotherapeutic drugs based on the action of hydrogel drug carriers has attracted wide attention, and we will summarize the function of immune therapy drugs combined with hydrogels (Table 1).

**TABLE 1:** Hydrogel-based immunotherapy.

Hydrogels	Therapeutic agents	Target cancers	Strategies	Ref
ED-OVA/CPG DNA hydrogel	OVA, CpG	EG7-OVA	ED-OVA combines with CpG to form injectable DNA hydrogel. It can effectively bind dendritic DC cells to improve antigen presentation efficiency and effectively inhibit the growth of primary and recurrent tumors	Umeki et al. (2015)
udOVA/Dgel	udOVA, CpG	EG7-OVA	OVA sustained-release system was developed from immune stimulated CpG DNA hydrogel. Inducing the expression of IL-6 mRNA in mouse skin can inhibit tumor growth	Umeki et al. (2015)
CHP/rmIL-12	IL-12	CSA1M fibrosarcoma	IL-12 is encapsulated in cholesterol containing pullulan (CHP) nano gel to ensure the slow release of cytokines and inhibit the growth of fibrosarcoma	Shimizu et al. (2008)
MS ∼ IL-15	IL-15	TRAMP-C2 prostate cancer	The hydrogel microspheres (MS) are covalently linked to IL-15 (MS ∼ IL-15) by a releasable linker to maintain IL-15 for a long time, to achieve the expansion of immune cells, inhibition of tumor growth	Hangasky et al. (2022)
aPDL1@BetP-gel	aPDL1	colorectal tumors	BetP based injectable nanofiber hydrogel for local delivery of aPD-L1 can be reprogrammed as an anti-tumor TME, which can achieve the control and continuous release of therapeutic drugs	Chen et al. (2020)
peptide hydrogels	aPD-L1, D-1MT	melanoma	A library of heat- and ROS-sensitive peptide hydrogels was developed for sustained delivery of aPD-L1 and IDO inhibitors and to modulate ROS levels in the tumor microenvironment to facilitate the release of immunotherapeutic drugs	Yu et al. (2018)
supramolecular hydrogels	DPPA-1 peptides, DOX	colon cancer	Injectable supramolecular hydrogel for topical delivery of DPPA-1 peptides and DOX. On the one hand, DOX can directly kill tumor cells or induce immunogenic cell death. On the other hand, DPPA-1 peptides can locally block the PD-1/PD-L1 pathway	Liu et al. (2021)
DC/alginate hydrogel	DCs		By mixing calcium-loaded alginate microspheres with soluble alginate solution and dendritic cells to form a hydrogel, host dendritic cells and a large number of T cells can be attracted at the injection site	Hori et al. (2008)
self-assembled poly(L-valine) hydrogel	TLR3 agonist, poly(I:C)	melanoma	Injectable self-assembled poly (L-valine) hydrogel is used as the delivery carrier of goods, which can cause strong cytotoxic T lymphocyte immune response and has good anti-tumor effect	Song et al. (2019)
peptide nanofiber hydrogels	DC, aPD-1, tumor antigens	EG7-OVA	The peptide nanofiber hydrogel containing DC, aPD-1 and tumor antigen was prepared to improve the immunotherapy of malignant tumors and show excellent anti-tumor immunotherapy efficiency	Yang et al. (2018)
SHV	Sev	melanoma and breast cancer	An injectable SEV-based hydrogel vaccine that multi-channel recruits and stimulates dendritic cells to enhance a specific immune response against tumors	Zheng et al. (2021)
oAd + DC/Gel	oAd, DCs	lung cancer	Gelatin-based hydrogels are used to co-deliver oAd and DC for sustained release of both therapeutics. The secretion of tumor-specific IFN-γ was significantly increased	Oh et al. (2017)
thermosensitive PCL-PEG-PCL hydrogel	GM-CSF, OVA		By encapsulating GM-CSF and OVA into a PCL-PEG-PCL matrix, a hydrogel with ideal local injection capability was constructed. The release of GM-CSF can enhance the accumulation of DC, further improve the efficiency of antigen uptake	Sun et al. (2018)
nHA/GM-CSF hydrogels	nHA, GM-CSF	melanoma	The co-encapsulation of nHA and GM-CSF into a biocompatible thermal PMG-PMG-PG-PGA hydrogel is conducive to the sustainable release of GM-CSF at tumor sites and enhances and prolongs anti-tumor immunity	Chen et al. (2022)
PEG-b-poly (L-alanine) hydrogel	GM-CSF, aCTLA-4/aPD-1	Melanom,4T-1 tumors	A PEG-b-poly(l-alanine) hydrogel was developed for combined delivery of oncology vaccines and dual immune checkpoint inhibitors	Song et al. (2019)

### Hydrogels based on a single immunotherapeutic agent are used in immunotherapy

**FIGURE 2:**
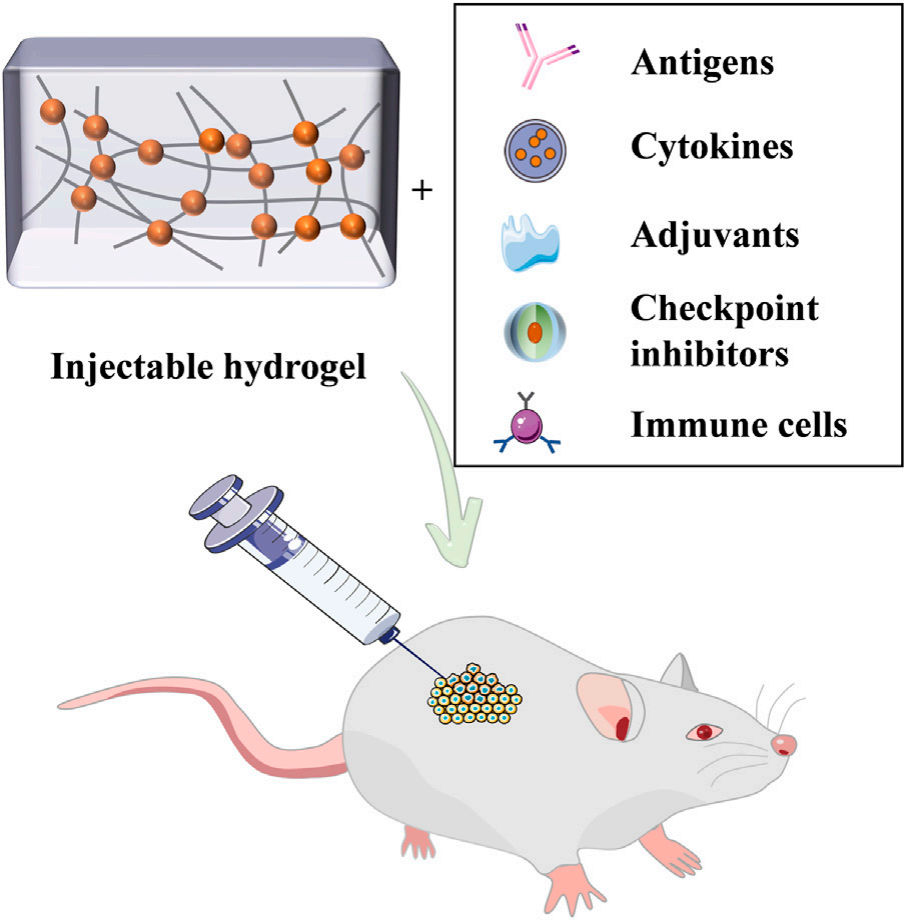
Injectable hydrogels based on immunotherapeutic agents for cancer immunotherapy.

Immunotherapeutic agents mainly include antigens, cytokines, adjuvants, checkpoint inhibitors, and immune cells, which can be individually loaded and transported by the hydrogel (Figure 2). Ovalbumin (OVA) can provoke robust OVA-specific immunity and has a good immune effect (Lee S.-J. et al., 2019; Habibi et al., 2020). According to the study, researchers devised a DNA hydrogel with auxiliary activity (Umeki et al., 2015). By conjugation of ethylenediamine (ED) and cationized OVA, complexes with hexapod structure DNA (ED-OVA) were developed with a slower release of OVA compared to unmodified OVA and induced higher IFN-γ production. Cytosine-guanine (CpG) dinucleotides is a positive immune adjuvant, and the integration of ED-OVA and immune-stimulating DNA injection hydrogels containing it is conducive to effective binding of DCs, thus improving the efficiency of antigen presentation.

Cytokines are a powerful weapon in immunotherapy, however, their half-life in patients is quite short. To overcome this major obstacle, Shimizu et al. loaded IL-12 into the cholesterol-containing pullulan (CHP) hydrogel to ensure its slow release of cytokines (Shimizu et al., 2008). A mouse model of CSA1M fibrosarcoma was established, and the hydrogel was injected subcutaneously into mice, which was found to cause a persisted increase in the concentration of IL-12 in serum. In addition, interleukin-15 (IL-15) is also a vital cytokine indispensable and has vast potential as tumor immunotherapy (Robinson and Schluns, 2017). Santi et al. prepared hydrogel microspheres (MS), which were connected to IL-15, to maintain IL-15 *in vivo* for a long time, namely ultra-long-acting IL-15 (Hangasky et al., 2022). It was found that the half-life of cytokines released from the library was prolonged, showing moderate tumor growth inhibition and effective and lasting anti-tumor activity.

Hydrogels load and transport immune checkpoint inhibitors to induce immune checkpoint blockade, which is a reliable immune tactic for the therapy of tumors. Chen and Liu’s group developed an injectable hydrogel based on betamethasone phosphate (BetP) for topical delivery of aPD-L1 (Chen et al., 2020). The study found that this steroid-based carrier-free system could reprogram the tumor microenvironment (TME) into anti-tumor TME, and it will continuously release aPD-L1 to combinational enhance the immunity system. The results showed that the gel administration of aPD-L1 greatly extended the retention time in the tumor. Gu’s group has developed a library of heat-sensitive and reactive oxygen species (ROS)-sensitive peptide hydrogels for sustained delivery of aPD-L1 and dextro-1-methyltryptophan (D-1MT), and modulates ROS concentration in the TME, facilitating the release of immunotherapeutic drugs that enhance efficacy against melanoma *in vivo* (Yu et al., 2018b). In addition, Yang’s group developed injectable supramolecular hydrogels for the topical delivery of DPPA-1 peptides and doxorubicin (DOX). DPPA-1 peptides can block the PD-1/PD-L1 pathway to enhance the T cell-mediated immune response (Liu et al., 2021). Similarly, Shi et al.’s study demonstrated that aPD-L1 and Dox combined hydrogel therapy with B16F10 melanoma models significantly restrained cancer progression and extended animal survival (Shi et al., 2021).

Dendritic cells (DCs) can activate the key APCs of immature T cells, which are effective initiators of the immune response (Banchereau et al., 2000; Steinman and Banchereau, 2007; Gardner et al., 2020). Treatment based on autologous dendritic cell injection has become a well-studied strategy (Bol et al., 2016; Santos and Butterfield, 2018; Bol et al., 2019; Perez and De Palma, 2019). Irvine et al. prepared an injectable hydrogel by combining alginate microsphere with DCs. The purpose was to achieve long-term storage of dendritic cells at a determined location, capture cytokines secreted by dendritic cells, and start the natural T cell immune response (Hori et al., 2008). The DCs vaccine is a promising and powerful immunotherapy for cancer, but it has drawbacks that cannot be ignored, the most critical one is that the transplanted DC dies, or is lost to a non-cancerous site, and rarely returns to the lymph nodes (Palucka and Banchereau, 2013; Abraham and Mitchell, 2016; Cornel et al., 2018), thus causing the T cell response is not strong enough. In recent years, there are still many studies on hydrogel-based dendritic cell vaccines. For example, Wang’s study showed that the hydrogel containing DC, aPD-1, and tumor antigen caused a robust immune response, showing excellent antitumor immunotherapeutic effect (Yang et al., 2018). Recently, Zheng and his colleagues devised a Sendai virus (SeV) based hydrogel vaccine (SHV), which can effectively inhibit the occurrence of melanoma (Zheng et al., 2021). In addition, to overcome the limitations of rapid spread and short half-life of therapeutic drugs at the tumor site (Chang et al., 2011; Liau et al., 2018; Goradel et al., 2021), Yun et al. used gelatin hydrogels to co-deliver onco-lytic adenoviruses AD. (Oh et al., 2017) This study discovered that, compared with single cure, the effect of this method on tumor growth was significantly increased. For hydrogels containing immune cells, some key questions should be considered to facilitate their potential applications. For example, these hydrogels require good biodegradability to achieve DC release.

The biological activity of a single immunotherapeutic drug is limited, so this strategy is not ideal for tumor treatment. Therefore, scientists have considered delivering multiple immunotherapeutic agents at the same time.

### Hydrogels based on multiple immunotherapeutic agents are used in immunotherapy

Recently, the PCL-PEG-PCL thermos-sensitive hydrogel encapsulated GM-CSF and OVA NPs to improve antigen absorption efficacy (Sun et al., 2018). It could trigger an effective immune response. In another study, Chen et al. co-encapsulated nano-hydroxyapatite (nHA) and GM-CSF into a biocompatible heat-sensitive hydrogel (Chen et al., 2022). Interestingly, the biological activity of nHA and GM-CSF can not only be very kept in hydrogel, but the addition of nHA can also weaken the erupt emancipate of GM-CSF, which is conducive to the continuously release of GM-CSF and realize enhanced and extended anti-melanoma immunity.

In another study, Wang et al. developed a PEG-b-poly (L-alanine) hydrogel, in which the tumor cell lysate, GM-CSF, and a dual immune checkpoint inhibitor (anti-CTLA-4/PD-1 antibodies) were easily loaded into (Song et al., 2019). It disclosed that the continuous release of cancer antigen and GM-CSF continuously recruited and stimulated DCs, and provoked robust T cell response. Immune checkpoint therapy boosted T cell response, and also upregulated the creation of IgG and cytokine secretion. Importantly, hydrogel combination therapy has a better immunotherapeutic effect on melanoma and 4T-1 tumor than using the vaccine alone or blocking immune checkpoints alone.

Overall, the co-delivery of hydrogel-based multiple immune therapeutics generally results in higher efficacy in treating tumors than a single drug (Blidner et al., 2020; Ramos-Casals et al., 2020). In addition, the design of such intelligently delivered composite hydrogels is often complex, which is a test for researchers.

## Chemotherapy-combinational immunotherapy

Chemotherapy is a cancer treatment in which various chemicals, such as chemotherapy drugs, are used to directly kill cancer cells (Anand et al., 2022). Chemotherapeutics is considered one of the most widespread approaches for treating malignancies (Liu et al., 2020; Xie et al., 2020; Yin et al., 2020). In addition, it has been shown that several chemotherapy agents that cause apoptosis of tumor cells can induce immunogenic cell death (ICD), thereby converting non-immunogenic tumors into immunogenic phenotypes, resulting in anti-tumor immune responses (Johnson et al., 2017; Wang Q. et al., 2018; Chattopadhyay et al., 2020; Wang-Bishop et al., 2020). However, chemotherapeutics is also associated with several general side effects unfortunately, such as bone marrow suppression and neurotoxicity, which often limit the effectiveness of chemotherapy drugs. Surprisingly, hydrogels can effectively reduce side effects by releasing drugs locally (Fan et al., 2019). Strategies using injectable hydrogels for local co-delivery of chemotherapy drugs and immunotherapy drugs have been applied to trigger long-term effective anti-malignant immune responses.

### Combination of chemotherapy with stimulant-mediated immunotherapy

Polymer-based hydrogels can be loaded with multiple chemotherapy drugs for cancer at the same time, and the efficacy can be improved through drug combinations. Melittin is a very potent anti-cancer agent, however, hemolysis is the main limitation of its application (Rady et al., 2017; Wang et al., 2022). To overcome this limitation, Yang et al. devised a peptide hydrogel encapsulated with DOX and Melittin-RADA32 (Jin et al., 2018). It can stimulate DCs, specifically consume M2 TAMs and recruit activated NK cells from tumors to further protect cells from residual tumors. It has significant anti-tumor effects on subcutaneous and metastatic tumors *in vivo*.

DOX is a potent anti-tumor cytotoxic chemotherapy drug and a representative ICD inducer that can lead to apoptosis of cancer cells (Hu et al., 2021). There has been a lot of research on the effective treatment of cancer using DOX as a chemotherapy drug loaded into hydrogels along with immunotherapeutic agents. Recently, Lv et al. devised an injectable hydrogel for the programmed delivery of DOX and cytosine-phosphate-guanine (CpG) nanoparticles guided by dual fluorescence imaging (Dong et al., 2020). CpG as an immune adjuvant can successfully stimulate an antitumor immune response by interacting with Toll-like receptor 9 (TLR9), and according to previous studies, the amalgamation of DOX and CpG is one of the most useful companions of chemotherapeutic immunotherapy (Kumagai et al., 2008; Wang Z. et al., 2017; Yu et al., 2017; Xia et al., 2018; Kheirolomoom et al., 2019). CpG self-crosslinked nanoparticles from hydrogels ensure long-term immunostimulant effects of dual continuous delivery systems. In addition to inducing apoptosis of tumor cells by doxorubicin, this hydrogel actively modulated the tumor microenvironment, which will construct a more favorable treatment response.

Imiquimod (R837) is also an immune adjuvant that activates TLRs and nuclear factor-kappa B (NF-κB) (Schon and Schon, 2007). Previously, Zhang et al. prepared a novel near-infrared (NIR) *in situ* resistant tumor vaccine by combining hyaluronic acid-modified polydopamine nanoparticles (HA-PDA NPs) with immunoadjuvants R837 and DOX into a thermosensitive hydrogel (Zhang et al., 2021). Due to the long retention time at the tumor site, after a single injection of near-infrared radiation, the NPs are endowed with multiple photothermal ablation characteristics. What’s more, this pattern enables it to stimulate DC maturation and trigger a robust anti-tumor immune response. Due to membrane permeability, nanoparticles can successfully enter the cancer tissues, generate tumor-associated antigens *in situ*, induce DC maturation and secrete related cytokines *in vitro*. Recently, Zhang et al. devised an injectable hydrogel loading DOX and R837 for the synergistic therapy of melanoma (Li J. et al., 2022). It efficiently inhibits melanoma growth and metastasis *in vivo* by DOX-based ICD, and R837-based immune responses are secreted by DC maturation, M1 type macrophage activation, TNF-α, and IFN-γ. This shows that this continuous release system can provide effective cancer treatment strategies, showing the potential of precise targeting tumor therapy.

Injectable hydrogels can be utilized to load and release chemotherapy agents and cytokines for chemoimmunotherapeutic. Chen et al. prepared an *in situ* forming hydrogel for the joint delivery of IL-15 and cisplatin (Wu et al., 2017). This peptide-based hydrogel can reduce systemic toxicity as a topical drug delivery vehicle. It can mediate the S-phase cell cycle block and increase CD8^+^ T cells. In another study in the same group, DOX-IL-2-IFN-γ co-loaded hydrogel was used for the chemical immunotherapy of melanoma. The antitumor effect was enhanced by expanding the proportion of apoptosis and G2/S phage cycle block (Lv et al., 2018).

In addition to the above stimulants, other immunomodulators also can be combined with hydrogels for amplified tumor immunotherapy. For example, In the article by Luan et al., the L-norvaline-based immunomodulatory gel is reported, which can effectively block the arginase 1 (ARG1) pathway (Ren et al., 2021). ARG1 can impair the synthesis of the T cell receptor (TCR) chain, contributing to reactive inactivated T cells (Bronte and Zanovello, 2005; Rodriguez et al., 2007; Geiger et al., 2016). The reactive cleavage of the hydrogel peptide bond ensures the controlled release of L-norvaline, thereby efficiently blocking the ARG1 pathway. DOX was further introduced to the hydrogel to trigger the ICD. It was found that this strategy showed a strong immunotherapeutic effect. Recently, Zhang et al. developed biomimetic nanobubbles loading DOX (DOX@LINV) for combined immunochemotherapy by blending synthetic liposomes with tumor-derived nanovesicles (TNVs) (Hu et al., 2021). Overall, DOX@LINV was found to enhance the penetration of effector immune cells and improve the immune suppressive TME.

### Combination of chemotherapy with checkpoint blockade immunotherapy

Injectable hydrogels are widely employed in the combined delivery of chemotherapy agents and immune checkpoint inhibitors for combined chemoimmunotherapy. The immune checkpoint inhibitors provoke a combined antitumor immune response by intervening with suppressive T cell signaling (Jenkins et al., 2018).

In cancer immunotherapy, it significantly enhances treatment response for immune checkpoint inhibitors to aim at the CTLA-4 and PD-1/PD-L1 axes (Littman, 2015; Klevorn and Teague, 2016; Robert, 2020; Naimi et al., 2022). For instance, Gu and others obtained ROS-responsive hydrogels by crosslinking polyvinyl alcohol (PVA) for co-delivery of chemotherapy gemcitabine and aPD-L1 (Wang C. et al., 2018). The designed hydrogels can be used to load and release therapeutic drugs when implanted at the tumor site because ROS is very abundant. This study showed that this type of gel scaffold promoted immunogenic tumor phenotyping and enhanced the anti-tumor response by local release of aPD-L1. In another study, a dual bioresponsive gel depot for co-deliver aPD1 and Zebularine is developed (Ruan et al., 2019). The results showed that this synergetic therapeutic improved tumor cells’ immunogenicity.

**FIGURE 3:**
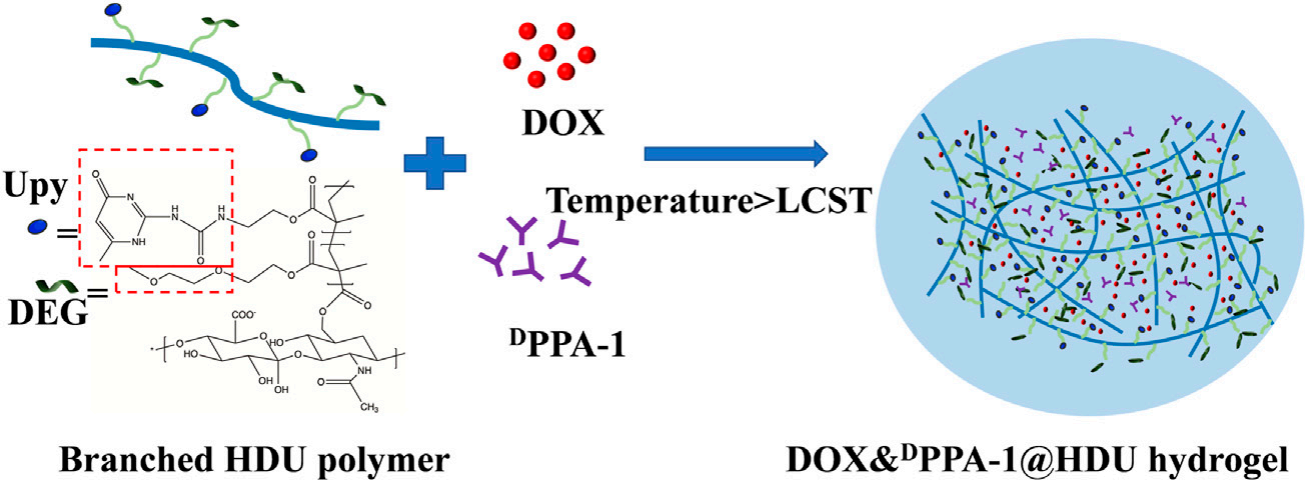
Schematic illustration of an injectable thermos-responsive hydrogel with ^D^PPA-1 and DOX.

Additionally, Yang et al. fabricated an injectable thermosensitive hydrogel loading DOX and PD-L1 agonist peptide (^D^PPA-1) (HDU hydrogels) (Liu et al., 2021). The HDU hydrogel steadily emitted encapsulated DOX and ^D^PPA-1 to stimulate an antitumor immune response. ^D^PPA-1 is based on the local blockade of PD-1/PD-L1 pathway, thereby enhancing the immune response mediated by T cells and reducing toxicity (Figure 3). In another study, Cui’s group established a prodrug hydrogel for locally delivering ICBs to further boost antitumor immunity (Wang et al., 2020). Their research results show that this hydrogel can be used as a continuous response emancipation library in TME, inducing powerful PD-1 to block the immune response.

Chemotherapy combined with injectable hydrogels containing immune checkpoint inhibitors is more effective than hydrogels loading a single immune checkpoint inhibitor (Table 2). Despite this, most synthetic hydrogels with complex structures should be carefully evaluated for long-term toxicity. In addition, the load ratio of agents should be elevated to achieve the maximum therapeutic benefit (Xie et al., 2021).

**TABLE 2:** Hydrogel-based chemoimmunotherapy.

Types	Hydrogels	Therapeutic agents	Target cancers	Strategies	Ref
Stimulator	MRD hydrogel	Melittin, DOX	melanoma	A DOX-loaded melittin-rada32 mixed peptide hydrogel was developed for chemoimmunotherapy against melanoma by actively regulating TMEs	Jin et al. (2018)
	αcyclodextrin/polyethylene glycol hydrogel	DOX, CpG	melanoma	The system consists of injectable hydrogels containing DOX and CpG NP. DOX induces tumor cell apoptosis, and CpG actively regulates tumor microenvironment	Dong et al. (2020)
	HA-PDA@IQ/DOX NPs	DOX, R837	breast cancer	By integrating HA-PDA NP with R837 and DOX into thermosensitive hydrogels. Near infrared radiation triggers a strong anti-tumor immune response	Zhang et al. (2021)
	MMP-2 sensitive hydrogels	DOX, R837	breast cancer	To construct matrix metalloproteinase sensitive hydrogel for the treatment of metastatic breast cancer. R837 triggers a strong immune response	Yan et al. (2020)
	injectable hydrogel loaded with DOX- and R837	DOX, R837	melanoma	The gel consists of four-arm PEGSH and PEGDA. DOX and R837 not only induce apoptosis, but also induce non-apoptotic cell death	Li et al. (2022a)
Checkpoint blockade	hydrogel containing IL-15/CDDP	Il-15, CDDP	melanoma	*In situ* formation of thermosensitive hydrogels based on mPEG-b-PELG. Co delivery of IL-15 and CDDP can reduce systemic toxicity and enhance anti-tumor immunity	Wu et al. (2017)
	PLN-PEG@DOX hydrogels	L-norvaline, DOX	melanoma	The hydrogel strategy of injection of l-norvaline immunomodulatory gel can effectively block the ARG1 pathway. DOX is further introduced into the hydrogel to trigger the ICD.	Ren et al. (2021)
	DOX@LINV	DOX	Melanoma, Lewis lung cancer, 4T1 breast cancer	By fusing artificial liposomes with tumor-derived nanovesicles, DOX-loaded biomimetic hybrid nanobubbles (DOX@LINV) were developed for combined immunochemotherapy	Hu et al. (2021)
	ROS-responsive hydrogels	GEM, aPD-L1	B16F10 melanoma, 4T1 breast tumors	By crosslinking PVA with ros-unstable linkers, the chemotherapy drugs GEM and aPD-L1 are co-transported	Wang et al. (2018a)
	Zeb-aPD1-NPs-gel	aPD1, Zeb	B16F10 melanoma	A bibioreactive gel library was designed that can react to acidic pH and ROS within TME to co-deliver aPD1 and Zeb	Ruan et al. (2019)
	HDU hydrogels	DOX, DPPA-1	CT26 colon cancer	Heat-sensitive hydrogels co-loaded with DOX and DPPA-1 were developed to stimulate anti-tumor immunity, enhance T cell-mediated immune responses, and minimize side effects	Liu et al. (2021)
	prodrug hydrogel	CPT, aPD1	GL-261 brain cancer, CT 26 colon cancer	A Prodrug hydrogel was developed for topical delivery of ICBs. Long-term release of CPT and aPD1 can cause strong and long-lasting systemic anti-cancer immunity	Wang et al. (2020)

## Radiotherapy-combinational immunotherapy

Radiation therapy(RT), a method of treating tumors by killing cancer cells with ionizing radiation of high-energy rays, is widely used in treatment because of its immediate and sustained response, accompanied by moderate inflammatory changes (Formenti and Demaria, 2013). X-rays, *γ* rays, and heavy ions are common types of radiation used in cancer radiation therapy (Baskar et al., 2012). Radiation has previously been presented to trigger an antitumor immune response, and radiotherapy has an immune-modulatory effect on the TME and adjacent normal tissues (Reits et al., 2006; Galluzzi et al., 2017; Guipaud et al., 2018; Cytlak et al., 2022). The influence of radiation therapy on the tumor immune system depends on the immune environment, dose, and the fractionation of radiation therapy (Romano et al., 2021). To overcome these problems, researchers have devised a protocol for loading radioactive material onto hydrogels (Table 3).

**TABLE 3:** Hydrogel-based radioimmunotherapy.

Hydrogel	Therapeutic agents	Target cancers	Strategies	Ref
^131^I-Cat/CpG/ALG	^131^I-Cat, CpG	breast cancer, prostate cancer	An *in situ* gel strategy to load^131^I radioisotope-labeled Cat onto ALG-based hydrogels to initiate effective radioimmunotherapy	Chao et al. (2018)
smart hydrogel	Aapt, CpG	colon cancer	A smart hydrogel was developed, which combines ALG with Aapt, hybridizes with CpG oligonucleotide, and forms hydrogel *in situ* after intratumoral injection	Sun et al. (2021)
Smac-TLR7/8 hydrogel	TLR7/8	B16 melanoma and 4T1 breast cancer	The toll-like receptor agonist TLR7/8a was coupled with a radiation-sensitive peptide hydrogel to modulate ITM, thereby regulating the repolarization of TAMs from M2 to M1 immunosuppressive tumor microenvironment	Zhang et al. (2022)

Liu et al. designed an *in situ* formed approach in which 131I radioisotope-labeled catalase (Cat) is encapsulated on alginate-based hydrogels to instigate radioimmunotherapy (Chao et al., 2018). Compared with brachytherapy, it shows a significantly superior therapeutic effect. Radioisotope-labeled enzymes are trapped within the tumor along with immunoadjuvants, consequently, radioisotope therapy (RIT), which is hypoxia-relieving, has an excellent local tumor-killing effect on the primary tumor.

Certain forms of chemotherapy drugs and ionizing radiation are known to induce ICD (Galluzzi et al., 2013). If immune adjuvants are present in the tumor, this anti-tumor immunity is amplified further (Steinhagen et al., 2011). However, since clinical chemotherapy/radiation therapy is often repeated in the form of low doses, it is unreasonable to administer immunoadjuvants to cancers at chemotherapy/radiation therapy dosage (Sun et al., 2021). Therefore, recently, Liu et al. devised an intelligent hydrogel that conjugates ALG to an ATP-specific aptamer, which is crossed with CpG oligonucleotides subsequently and forms an alginate-based hydrogel *in situ* during the intratumoral injection (Sun et al., 2021). By irradiating CT26 mouse colon cancer cells with an X-ray dose gradient, it was found that intracellular ATP concentrations increased significantly. This suggests that X-rays can trigger the release of ATP while inducing ICD in tumor cells. ATP then competitively binds to apt to trigger CpG release, thereby enhancing anti-tumor immunity after ICD induction therapy. It was found that the *in situ* smart hydrogel based on ALG significantly enhanced anticancer immune responses and boosted the overall therapeutic effect of repeated chemotherapy combined with immunotherapy. Thus, this smart hydrogel can release immune adjuvants synchronized with radiotherapy, and other experiments have also proved that this smart hydrogel achieved a significant synergistic reaction and inhibited tumor regeneration and metastasis.

**FIGURE 4:**
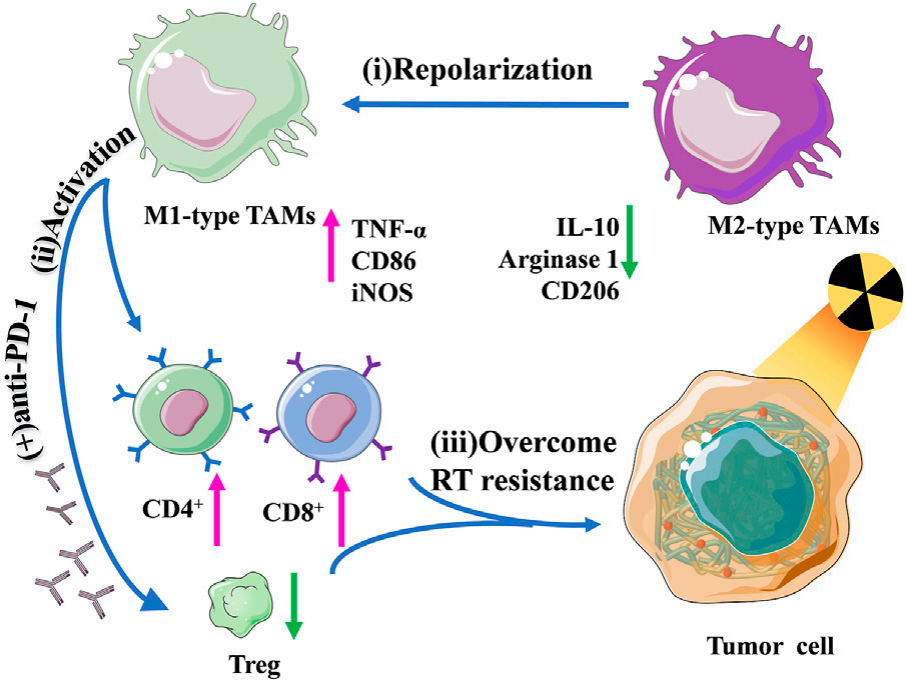
Schematic illustration of the regulation of macrophage repolarization to overcome radiation resistance by the Smac-TLR7/8 hydrogel.

Another study of radiotherapy combined with immunotherapy was recently published. Liu and others modulated immunosuppressive tumor microenvironment (ITM) and overcome radioactivity by coupling the Toll-like receptor agonist TLR7/8a with a radiation-sensitive hydrogel (Smac-TLR7/8 hydrogel) to adjust TAMs repolarization from M2 type into M1 type (Zhang et al., 2022). In the C57BL/6 mouse model, after treatment with reasonable doses of *γ* irradiation, it was found that TMs could be repolarized to the M1 type by stimulating the NF-κB pathway, which significantly improved the radiotherapeutic effect on mouse tumors. What’s more, macrophage repolarization induced immune responses, promoted the recruitment of tumor-infiltrating lymphocytes, and decreased regulatory T cells, effectively alleviating the ITM. This study showed that this novel strategy, both hydrogel-based radiation therapy combined with immunotherapy, reconstructed ITM through repolarizing TAMs, effectively improving RT efficacy, and overcoming radioactivity (Figure 4).

## Phototherapy-combinational immunotherapy

Photosensitizer-based photoactivation therapy has been determined as a harmless way of tumor ablation for many tumor indications. There are mainly two methods: photodynamic therapy (PDT) causing local chemical damage, and photothermal therapy (PTT) causing local thermal damage (Li X. et al., 2020). In addition, PTT and PDT perform a vital part in activating anti-tumor immune responses because they can initiate ICD, thereby increasing tumor immunogenicity (Ng et al., 2018; Li X. et al., 2020). The use of nanomaterials as carriers combined with phototherapy and immunotherapy can magnify the immune response and enhance the curative effect, therefore, several hydrogels have been exploited for phototherapeutic combination immunotherapeutic for the treatment of cancer.

### Photothermal therapy (PTT) -combinational immunotherapy

PTT is a hopeful approach that employs ingredients with extraordinary photothermal conversion efficiency to transform illumination energy into heat (Huang et al., 2006; Yang et al., 2010; Huang P. et al., 2019). The PTT-induced hyperthermic effect defeats cancer cells by damaging cell membranes and generating DNA injury, thereby causing cell necrosis, cell apoptosis as well ICD (Hildebrandt et al., 2002; Li Y. et al., 2020; Gao et al., 2020). By filling it and immunotherapeutic agents into hydrogel carriers, PTT combination immunotherapy can be achieved.

PTT has been reported to promote the discharge of the protein antigens from tumor by inducing immunogenic cell death, which exposes large amounts of autoantigens after PTT treatment, thereby enhancing a robust immune response (Ng et al., 2018; Wang et al., 2019). Wang et al. presented a heat-sensitive nanogels loading MnO_2_ NPs to activate anti-tumor immune responses (Fan et al., 2021). In particular, the catechol group is introduced into the structure of the heat-sensitive nanogel, and its strong adhesion gives it the capability to capture the antigens produced by PTT. After intertumoral injection of hydrogel, it continuously releases antigens to realize improved and extended immune stimulation, inducing both strong cytotoxicity and ICD. It indicated that the use of PTT based on injection of adhesive hydrogel resulted in complete ablation of the solid tumor and efficient inhibition of distal and regenerated cancer cells.

The combination of photothermal effect and immune adjuvant will result in a more effective immunotherapeutic effect, there has been a lot of research on PTT-combination immunotherapy based on hydrogels loaded with immunoadjuvants (Guo et al., 2014). Nishikawa’s group devised a compound immune-stimulatory hydrogel consisting of hexapod DNA with CpG sequence and Au NPs (Yata et al., 2017). Laser irradiation l leads to hexapods release, which effectively stimulates the stimulation of macrophages and DCs, and stimulates the emancipation of pro-inflammatory cytokines.

In another study using CpG as an immune adjuvant, Lv and coworkers proposed a conjugated hydrogel with CpG self-crosslinked NPs and IR820 for combined PTT and immunotherapeutic (Dong et al., 2019). It can advance continuous release by blocking rapid degradation of CpG, with CpG-mediated immunostimulatory effects of TLR-9 activation. In the B16 melanoma mouse model, after injection of CpG NPs/IR820 hydrogel and irradiation treatment, DC maturation and CD8^+^ T cell activation were moderately enhanced, and the anti-tumor immune effect was enhanced. More specifically, auto-fluorescent CpG NPs/IR820 hydrogels do not need additional fluorescence and are used for image-guided synergistic PTT and immunotherapeutic using a dual fluorescence imaging technique. It provides a platform full of possibilities and hopes for precision cancer diagnosis and therapy. Furthermore, Wei et al. designed a local injectable platform to achieve combined PTT and immunotherapy, which was formed by the photothermal agent Indocyanine Green (ICG), the TLR-7/8 agonist (R848), and the TLR-9 agonist CPG ODN (Jia et al., 2020). NIR triggered the thermally responsive hydrogel PLEL to release the immune components CPG ODN and R848, which, together with tumor-associated antigens, induce effective and sustained anti-tumor immunity as carcinoma *in situ* vaccines for postoperative immunotherapy.

Apart from immune adjuvants, strategies for immune checkpoint blocking can also be introduced into injectable hydrogels for photothermal immunotherapeutics. As Sun et al. proposed an all-in-one and fully controlled combination strategy, NIR photothermal agents IR820 and aPD-L1 were loaded into a lipid gel library with excellent thermally reversible gel-sol phase transition properties to achieve native symbiotic PTT-assisted immunotherapeutic (Huang L. et al., 2019). This study shows that symbiotic mild photothermal-assisted immunotherapy is an operational and potential approach for curing “cold” tumors. Other than that Li et al. proposed a personalized cancer vaccine (PVAX) for postoperative immunotherapeutics, by loading tumor cells with a BRD4 inhibitor and ICG into the hydrogel-based matrix (Wang T. et al., 2018). PVAX can advance the growth of DCs, and trigger tumor penetration of cytotoxic T lymphocytes.

PTT can also be used in combination with chemotherapy for cancer immunotherapy. For instance, Liu’s group developed a Ag_2_S QD/DOX/Bestatin@PC_10_ARGD peptide gel for breast cancer treatment as a lasting-release material (Hou et al., 2020). The experiments were carried out and it presented that the laser-irradiated hydrogel could stimulate anti-tumor immunity, relieve tumor pressure, and inhibit primary tumor and lung metastasis. At the same time, the safer low-temperature several laser irradiation approaches showed more effective tumor-killing performance. In summary, the strategy of combining PTT with other therapies based on injectable hydrogels effectively improves the effect of cancer treatment and is a promising and potential option.

### Photodynamic therapy (PDT)-combinational immunotherapy

Lately, PDT has fascinated widespread notice owing to its little toxicity, great spatiotemporal selectivity, minimally invasive, and considerable therapeutic effect (Zhang and Li, 2018; Shu et al., 2021). It can not only exactly destroy cancer cells, but also initiate ICD, providing anti-tumor immunity. Regrettably, the inherent nature and complex limitations of TMEs drastically reduce the efficacy of PDT (Ji et al., 2022). Emerging smart polymer hydrogels have been developed, loaded with immunotherapeutic agents through the combined action of PDT and immunotherapy, cleverly modulating the pharmacokinetics of drugs and TME, thus improving the anti-tumor efficacy.

Oxygen is essential for PDT to perform therapeutic roles, but the hypoxic TME present in most solid tumors will significantly restrain the efficiency of PDT (Yang et al., 2017; Min et al., 2021). To relieve hypoxic TME, Liu et al. used PEGDA as a polymer matrix to construct a light-triggered gelation procedure encompassing catalase modified by the photosensitizer (Meng et al., 2019). First, the photosensitizer Chlorin e6 (Ce6), which is extensively used in clinical, is coupled with catalase (CAT), and then the resulting coupling is mixed with the polymer matrix PEGDA and R837-loaded PLGA nanoparticles (RPNPs) as immune adjuvants. The retained CAT in the hydrogel reverses immunosuppressive TME by decomposing endogenous H_2_O_2_ of tumors to produce O_2_ leading to sustained remission of tumor hypoxia. Subsequently, tumor cell fragments after PDT-triggered ICDs can pretend TAAs and elicit a strong anti-tumor immune response alongside with RPNPs. In addition, this hybrid hydrogel can offer the chance to continually stimulate the immune response by triggering PDT through several times of lighting, which contribute to an extensively improved immune response. Studies have also found that further combination with a CTLA-4 checkpoint blockade could inhibit the evolution of distant metastatic cancers.

Sun’s team developed a continuous luminescent immune hydrogel by introducing a persistent luminescent material (PLM) and immunoadjuvant R837 into alginate-Ca^2+^ to convert solids into gels (Shu et al., 2021). PLM is a unique property that can stimulate continuous and repetitive PDT (Lécuyer et al., 2016; Wang J. et al., 2017; Wu et al., 2020; Wang et al., 2021). PLM, R837 and alginate-Ca^2+^ solutions were mixed to synthesize a rechargeable immune hydrogel (PRA) with a homogeneous structure. The designed PRA has great biocompatibility and excellent injectability, and can be effortlessly inserted into the tumor site, which the continuous luminescence efficiency of PLM can reach 100%. The experiments have exposed that DCs are activated, resulting in increased emission of TNF-α and IL-6, and a significant amplification of the anti-cancer immune response. It showed that the reliable anti-tumor immune effect is obtained by hydrogel-based PDT synergistic immunotherapy.

Recently, Li and others reported the use of NIR dual-response precursor hydrogels for synergistic immunotherapy for cancer (Ding et al., 2022). This prodrug hydrogel is formed by calcium-induced iron oxide (Fe_3_O_4_) nanoparticles modified with the photosensitizer protoporphyrin IX (PpIX) and aPD-L1 prodrug nanoparticles crosslinked through ROS-responsive linkers. This hydrogel enabled the on-demand discharge of aPD-L1 during photoactivation. This type of prodrug hydrogel could stimulate ICD by mediating photokinetic and chemokinetic therapy and expand the efficiency of aPD-L1-mediated immune checkpoint blockade. Together, this study provided a double-response hydrogel strategy for precise tumor immunotherapy.

**FIGURE 5:**
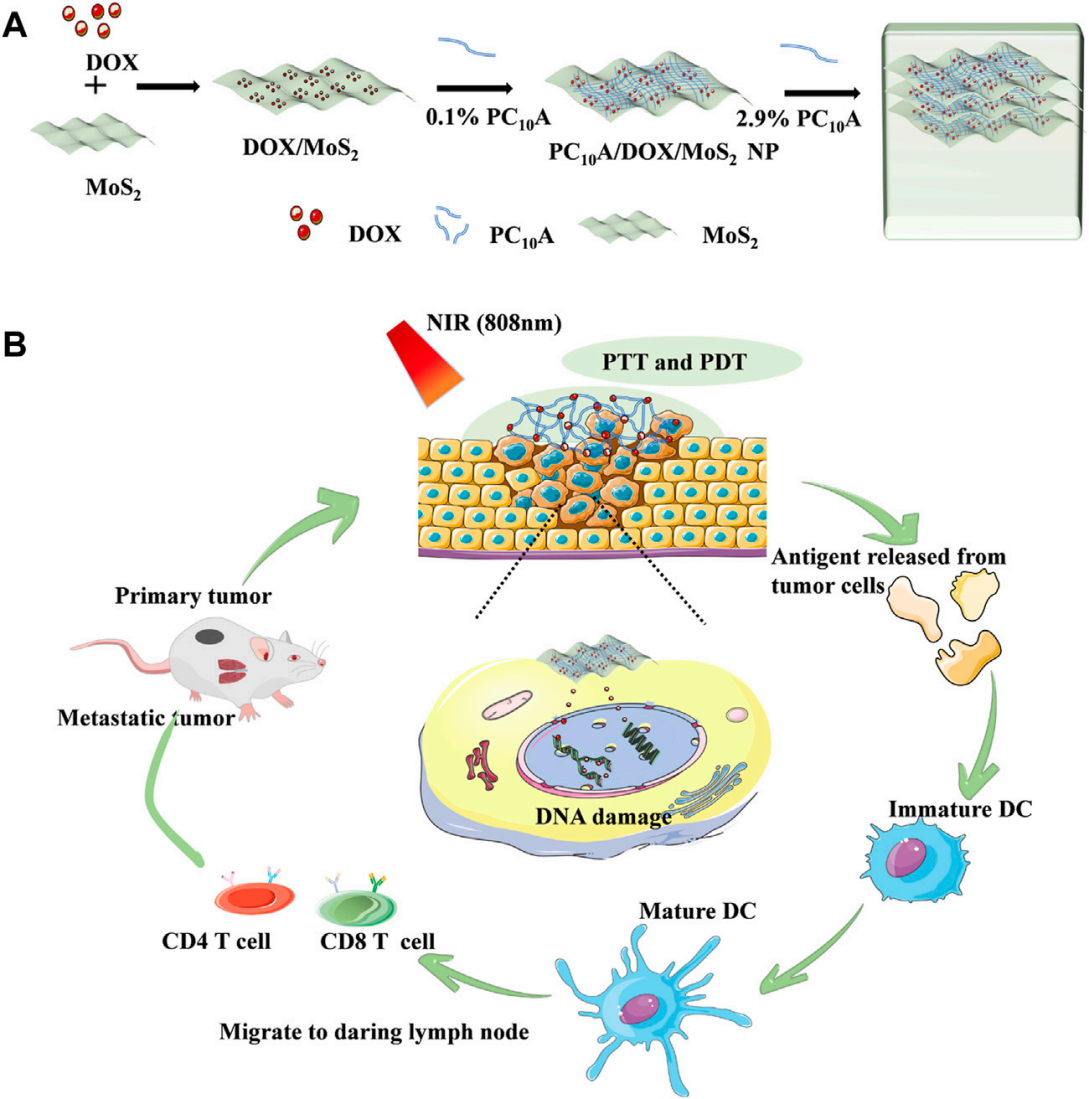
Schematic illustration of injectable hydrogels with both PTT and PDT effects based on DOX, peptide PC_10_A and MoS_2_ nanosheets. **(A)** Schematic illustration of the preparation for PC_10_A/DOX/MoS_2_ hydrogel; **(B)** Chemo-photothermal-photodynamic therapy.

Sometimes, two combinations of therapies still do not meet the therapeutic effect, so researchers try to use multiple treatments to enhance the efficiency and achieve the purpose of treatment. For example, Cao and Liu et al. devised and formulated a hydrogel based on DOX, polypeptide PC_10_A, and MoS_2_ nanosheet (Figure 5). (Jin et al., 2020) Firstly, positively charged DOX and negatively charged PC_10_A are filled on the exterior of MoS_2_ nanosheets by electrostatic adsorption, and then hybrid PC_10_A/DOX/MoS_2_ NPs are ‘dissolved’ in PC_10_A hydrogel to form an injectable hydrogel. The experiments showed that it has excellent photothermal efficiency. MoS_2_ nanosheets are materials with both PTT and PDT effects. It was found that the amount of DC, CD4^+^ T cells, and CD8^+^ T cells increased, and the combination of these treatments greatly increased the number of tumor cell apoptosis. In conclusion, this study showed that the combination therapy meaningfully enhanced the impact of tumor inhibition and inhibited the development of primary tumors and remote metastasis.

In summary, phototherapy-activated ICDs can stimulate immune responses, hydrogel-mediated combination phototherapeutic and immunotherapeutic generally provide better efficiency than hydrogel-mediated immunotherapeutic (Table 4). Compared to hydrogel-mediated combination chemoimmunotherapy, this approach likewise showed excellent controllability in space and time.

**TABLE 4:** Hydrogel-based photoimmunotherapy.

Types	Hydrogels	Therapeutic agents	Target cancers	Strategies	Ref
PTT	Heat-sensitive nanogels	MnO2NPs	4T1 breast cancer	An injectable adhesive hydrogel based on heat-sensitive nanogels, loaded with MnO2NPs, acts as PTA to stimulate PTT-induced anti-tumor immune responses	Fan et al. (2021)
	gold nanoparticles-DNA hydrogel	gold nanoparticle, CpG	EG7-OVA	A complex immunostimulatory DNA hydrogel consisting of a hexapod structure of DNA with CpG sequences and gold nanoparticles	Yata et al. (2017)
	CpG NPs/IR820 hydrogels	CpG, IR820	B16 melanoma	IR820-conjugated hydrogels loaded with CpG self-crosslinked nanoparticles were designed to play in combination with photothermal immunotherapy	Dong et al. (2019)
	RIC NPs @ PLEL hydrogel	ICG, R848, CPG- ODN	4T1 breast cancer	A thermosensitive PLEL hydrogel composed of ICG, R848 and CPG ODN was designed to achieve synergistic photothermal immunotherapy	Jia et al. (2020)
	lipid gel library	IR820, aPD-L1	breast cancer, melanoma	IR820 and aPD-L1 are loaded into the lipid gel library to achieve mild photothermal adjuvant immunotherapy	Huang et al. (2019)
	PVAX	JQ1, ICG	4T1 breast cancer	A personalized cancer vaccine for postoperative immunotherapy developed by co-loading tumor cells with JQ1 and ICG with a hydrogel matrix	Wang et al. (2018b)
	Ag_2_S QD/DOX/Bestatin@PC10ARGD hydrogel	Ag_2_S QD,DOX, Bestatin	breast cancer	Developed a Ag_2_S QD/DOX/Bestatin @PC_10_ARGD genetically engineered peptide hydrogel, which can effectively treat breast cancer under laser irradiation	Hou et al. (2020)
PDT	Ce6-CAT/RPNPs/PEGEDA	Ce6, CAT, R837	breast cancer	A photo-triggered *in situ* gel system, including photosensitizer modified catalase and PEGDA diacrylate, can induce strong immune effects	Meng et al. (2019)
	PRA	PLM, R837		A continuous luminescent immune hydrogel was developed by introducing a persistent luminescent material and an immunoadjuvant into alginate-calcium to convert solids into a gel	Shu et al. (2021)
	prodrug hydrogel	Fe_3_O_4_, PpIX, aPD-L1	4T1 breast cancer	NIR dual-reactive precursor hydrogels formed by crosslinking of photosensitizer calcium-induced iron oxide nanoparticles with PpIX and aPD-L1 prodrug nanoparticles through ROS response linkers	Ding et al. (2022)
	PC_10_A/DOX/MoS_2_ hydrogel	DOX, MoS_2_	breast cancer	An injectable hydrogel based on genetically engineered peptide PC_10_A, MoS_2_ nanosheets and DOX was designed and prepared for photoimmunotherapy	Jin et al. (2020)

## DNA hydrogel and cancer immunotherapy

Gene therapy is a developing targeted therapy approach for the treatment of malignant tumors. It aims to prevent tumor proliferation and metastasis by specifically targeting genes closely related to tumor genesis, progression, and prognosis. However, currently, there are few studies about hydrogels for gene therapy combined with immunotherapy.

**FIGURE 6:**
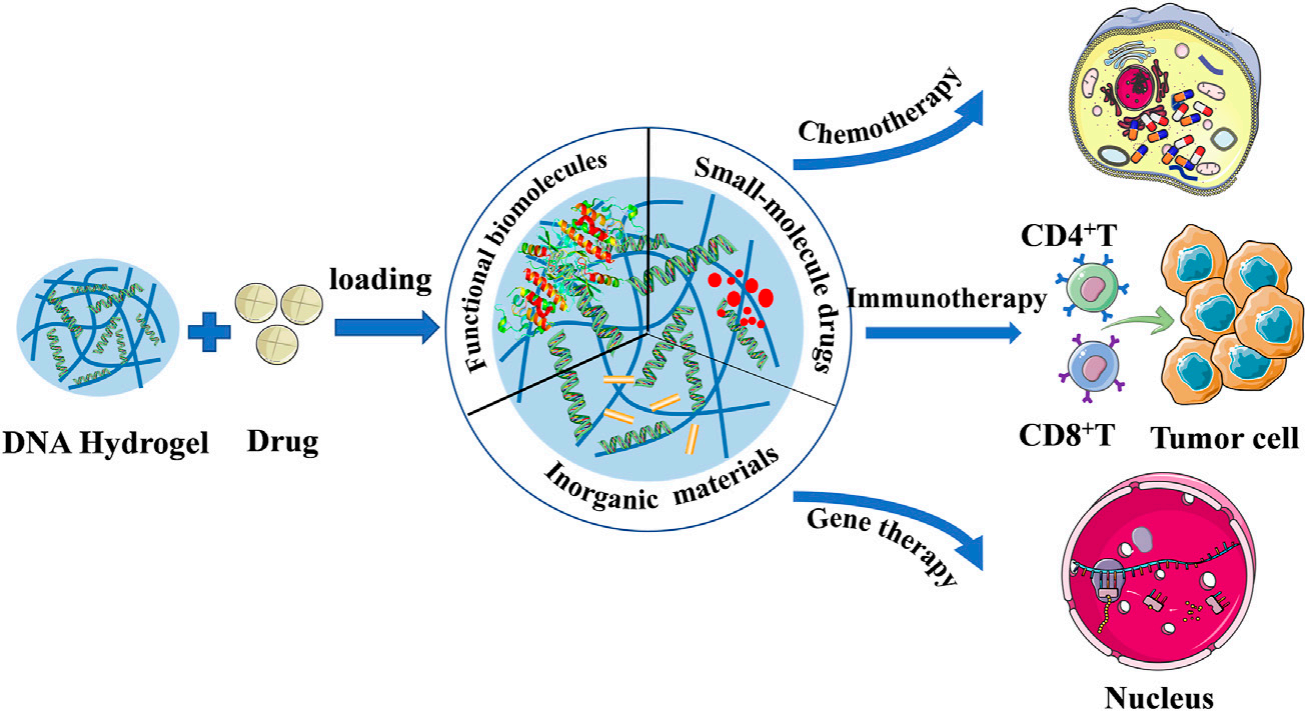
DNA based hydrogels for cancer therapy.

The Achilles heel of gene therapy is gene conveyance, which has been difficult to achieve the desired success as a result of the shortage of an operational delivery system, prolonged-expression, and host immune response (Verma and Somia, 1997). However, today, the research on excellent delivery systems is more extensive and deep, which can help overcome this problem and make the future of gene therapy full of possibilities. Current great advances in the development of DNA-based nanostructure technology, it has several advantages as a drug delivery procedure, such as its high water-solubility, stability, and biodegradability (Chhabra et al., 2010; Nishikawa et al., 2010; Nishikawa et al., 2011; Rangnekar and LaBean, 2014). DNA hydrogels have the advantages of outstanding biocompatibility, adjustable mechanical properties, controllable phase transitions, and simple preparation, and have broad prospects as suitable carriers (Mo et al., 2021). As an excellent drug delivery platform, DNA hydrogel can deliver different drugs, including functional macromolecules, small molecule drugs and inorganic materials, and plays an important role in cancer chemotherapy, immunotherapy and gene therapy (Figure 6). (Mo et al., 2021) Several studies of immunotherapy based on DNA hydrogel delivery will be presented next.

Nishikawa and others fabricated a novel greatly immunostimulatory DNA component by combing six very effective CpG motifs into X-DNA and formulated hydrogel by ligating with DNA ligase, and doxorubicin(DXR) was inserted into DNA subsequently (Nishikawa et al., 2011). This kind of DNA was found to be more operational than its component or CpG counterpart. The anti-tumor ability of the synthesized hydrogel was examined in colon 26/Luc cells, and CpG transmitted an immune stimulation signal to TLR9-positive immune cells to slow-release DXR.

Li et al. demonstrated a DNA supramolecular structure hydrogel vaccine (DSHV) consisting of the TLR9 agonist unmethylated CpG, and tetanus toxoid-derived amino acid peptide P30 co-loaded into the DNA hydrogel network (Shao et al., 2018). The amino acid peptide P30 is a helper T cell epitope that has been shown to conjugate to the MUC1 glycopeptide antigen and can cause a super strong immune response and induce a stronger immune response and is considered a very hopeful therapeutic antitumor vaccine (Cai et al., 2013). This study found that the DSHV system can usefully recruit and trigger APCs. The process *in vitro* can be imagined by fluorescence microscopy. *In vivo* experiments, by intraperitoneal or subcutaneous injection of BALB/c mice, it was found that the DSHV system can simulate the role of lymph nodes (LNs). At the same time, antigenic peptides exert a strong immune effect and synergistic anti-tumor immune effect in DNA supramolecular hydrogel.

It’It is well known that CRISPR-associated protein 9 is a potential genome editing tool, which is a complex of RNA-guided DNA endonuclease single-stranded guide RNA (sgRNA) and can enable Cas9 to cut DNA sequences precisely (Anders et al., 2014; O'Connell et al., 2014; Jiang and Doudna, 2017). In a particular study, researchers explored the usage of Cas9/sgRNA to regulate the emancipation of DNA aptamers from the hydrogel. Wu’s group used Cas9/sgRNA’s specific double-stranded DNA editing capability which can emancipate PD-1 aptamers programmatically (Lee J. et al., 2019). PD-1 DNA aptamers act as immune checkpoint inhibitors to generate hydrogels comprising it and sgRNA targeting sequence by rolling loop amplification (RCA). When mingled with Cas9/sgRNA, it may lose gel property and accurately release PD-1 aptamer sequences, and gel electrophoresis confirmed that this program release mode can successfully stimulate the occupation of splenocytes to secrete cytokines. Furthermore, molecular imaging showed higher anti-tumor effects and survival rates. It indicated that the combination therapy of the DNA-based hydrogel plus Cas9/sgRNA exerted anti-cancer effects by supporting the initiation of the immune system. This study showed that hydrogel has powerful anti-cancer immunotherapy potential, and also demonstrates a promising future in which gene editing plays a therapeutic role in synergistic with immunotherapy to treat cancer.

The results of these studies show that DNA can be operated as a transmission system of immunostimulatory signal and antitumor agents, and has a promising application value. It is supposed that the cooperation of gene therapy and immunotherapy based on hydrogel will achieve a big step forward and help human beings climb the mountain of “cancer”.

## Conclusion and outlook

In this article, the research progress of injectable hydrogel in cancer immunotherapy was discussed. The biocompatibility, injectability, degradability, easy encapsulation, stimuli-responsive, and local sustained release of hydrogels make them promising cancer immunotherapy materials (Bu et al., 2019). Single or multiple cancer immunotherapies and combination therapies can trigger and enhance a strong immune response, which can effectually restrain the growth of solid tumors. However, there are still some unavoidable problems with this treatment strategy. First, individual heterogeneity of patients may be the cause of cancer treatment failure, so there is also a need to strengthen research on the immune system, innovate and optimize injectable hydrogels to achieve personalized ideal treatment. Secondly, synthetic polymer-based hydrogels have the potential to cause chronic inflammation and immune response, and it is also necessary to continuously monitor the toxicity and biocompatibility of synthetic hydrogels and their degradation products (Merkle, 2015; Piluso et al., 2017). Thirdly, how to accurately inject hydrogels into deep tumors is a huge challenge. Multiple imaging techniques for deep tumor administration should be developed to guide the injection process. At last, the dosage and release rate of immunotherapeutic agents administered *in vivo* still needs to be further explored, and in clinical practice, the dose and rate of administration should be strictly controlled. Fifthly, the vast majority of existing research is based on mouse models, to achieve clinical application and experiments on other large animal models.

In short, hydrogels are materials with excellent prospects for cancer immunotherapeutic, and although there are many challenges to be faced in the future, we believe that with the unremitting efforts of scientists, we will be able to develop an ideal hydrogel delivery system to maximize the immune effect to treat cancer, and this biggest “threat” to human life and health will 1 day be defeated.
